# Successful use of obinutuzumab in focal segmental glomerulosclerosis with inadequate response to rituximab: a case report

**DOI:** 10.3389/fneph.2026.1772736

**Published:** 2026-03-03

**Authors:** Andreia Rita Henriques, João Venda, Emanuel Ferreira, Nuno Oliveira, Helena Sá

**Affiliations:** 1 Nephrology Department, Hospitais da Universidade de Coimbra, Unidade Local de Saúde de Coimbra, Coimbra, Portugal; 2 University of Coimbra, Faculty of Medicine, Coimbra, Portugal

**Keywords:** focal segmental glomerulosclerosis (FSGS), minimal change disease, nephrotic syndrome (NS), obinutuzumab, podocytopathy, rituximab inadequate response

## Abstract

**Introduction:**

Podocytopathies such as minimal change disease (MCD) and focal segmental glomerulosclerosis (FSGS) remain therapeutic challenges in adults. Although corticosteroids and rituximab (RTX), a chimeric anti-CD20 monoclonal antibody, are effective in most patients, up to 10% show resistance or relapse despite B-cell depletion. Obinutuzumab (OBZ), a humanized type II anti-CD20 monoclonal antibody, achieves deeper and more sustained B-cell depletion and may overcome RTX inadequate response.

**Case report:**

A 33-year-old woman presented with nephrotic syndrome (proteinuria 7.1 g/24 h, serum albumin 2.6 g/dL, preserved renal function). Kidney biopsy revealed primary FSGS. She achieved only partial remission with corticosteroids and cyclosporine. RTX (1 g × 2 doses) induced transient peripheral B-cell depletion but no complete remission. A second biopsy excluded chronic changes, and genetic testing for hereditary podocytopathy was negative. Thus 67 weeks after diagnosis and initial treatment with persistent proteinuria > 1g/24 h and hypoalbuminemia, the patient received OBZ (1 g × 2 doses, two weeks apart). Two months later, she achieved complete remission (proteinuria 0.2 g/24 h, serum albumin 3.7 g/dL), with sustained B-cell depletion and no adverse events. A repeat administration of OBZ (1 g) was performed 10 months later due to B-cell repopulation, rising proteinuria (0.6 g/24 h), and mild hypoalbuminemia (serum albumin 3.4 g/dL), successfully re-inducing complete remission (proteinuria 0.2 g/24 h, serum albumin 3.8 g/dL).

**Discussion:**

This case illustrates the potential of OBZ as an effective therapeutic option in podocytopathies with RTX inadequate response. The superior efficacy of OBZ may result from enhanced antibody-dependent cellular cytotoxicity, depletion of tissue-resident B cells, and reduced immunogenicity compared with RTX. OBZ may thus offer an alternative in refractory MCD/FSGS.

## 1 Introduction

The management of podocytopathies such as minimal change disease (MCD) and primary focal segmental glomerulosclerosis (FSGS) remains a significant therapeutic challenge in adults. Corticosteroids are the mainstay of treatment, with initial response rates of approximately 80–95% in MCD and 50–60% in FSGS. However, sustained remission is achieved in lesser than 45% of these patients (1). This has led to the exploration of alternative immunosuppressive strategies, including B-cell–targeted therapies.

Rituximab (RTX), a first-generation chimeric anti-CD20 monoclonal antibody, has demonstrated efficacy and safety in frequently relapsing or steroid-dependent podocytopathies, as shown in recent studies such as RITERM (2, 3). Nonetheless, relapses remain frequent, occurring after B-cell recovery, during incomplete or transient depletion, and even in the setting of ongoing peripheral B-cell suppression. In addition, approximately 5–10% of patients exhibit resistance to RTX (4, 5).

Obinutuzumab (OBZ) is a third-generation, type II, glycoengineered, humanized IgG1κ anti-CD20 monoclonal antibody developed to overcome RTX resistance, initially in B-cell malignancies. Compared with RTX, OBZ exhibits enhanced antibody-dependent cellular cytotoxicity or phagocytosis due to increased affinity for FcγRIIIa, and is less dependent on complement-mediated cytotoxicity, resulting in more potent direct cell death (6, 7). It also induces deeper and more sustained depletion of both circulating and tissue-resident B cells (6, 7), and demonstrates superior cytotoxicity toward naïve and switched memory B cells - subsets implicated in relapse after RTX therapy (8, 9). Moreover, OBZ may target residual CD20+ T cells, a subset group of T cells implicated in the pathogenesis of MCD and FSGS (10).

Emerging evidence suggests that OBZ provides a more potent and durable B-cell–depleting effect than RTX. However, its role in adult patients with RTX inadequate response MCD or FSGS remains largely unexplored. We present a case of an adult patient with RTX inadequate response FSGS who achieved complete remission following OBZ therapy.

## 2 Case report

A 33-year-old caucasian woman with no significant past medical history presented with a sudden onset of peripheral and periorbital edema, with normal blood pressure (120/70 mmHg). Laboratory evaluation revealed a serum creatinine of 0.68 mg/dL (estimated glomerular filtration rate [eGFR] 118 mL/min/1.73m2 by CKD-EPI), hypercholesterolemia, serum albumin (SAlb) of 2.6 g/dL, and a 24-hour proteinuria of 7.1 g without hematuria or leukocyturia. Given the nephrotic syndrome, further investigations were performed. Complement levels were normal, cryoglobulins, phospholipase A2 receptor (PLA2R) and thrombospondin type 1 domain-containing 7A (THSD7A) antibodies were negative. Screening for other autoimmune or infectious diseases was unremarkable, and no monoclonal proteins were detected. Kidney ultrasound showed normal kidneys, and no remarkable findings were seen on a thoracoabdominal pelvic CT scan.

A kidney biopsy was performed. Histological examination of 36 glomeruli revealed one globally sclerosed glomerulus and one glomerulus with a segmental sclerotic lesion, classified as focal segmental glomerulosclerosis (FSGS), not otherwise specified (NOS) variant. There was mild mesangial hypercellularity, without endocapillary or extracapillary proliferation. The basement membranes were normal. Interstitial fibrosis/tubular atrophy (IFTA) was mild (approximately 12%), and mild arteriosclerosis was present. Immunofluorescence showed focal and segmental mesangial positivity for IgM and C3; IgG, IgA, C1q, κ, and λ were negative.

A diagnosis of primary FSGS was established. Supportive management was initiated, including sodium restriction (2 g/day), diuretics, lipid-lowering therapy, and blood pressure. An angiotensin receptor blocker (losartan 25mg daily) was attempted but discontinued because of symptomatic hypotension. Blood pressure subsequently remained well controlled (<120/80 mmHg) without antihypertensive therapy. Immunosuppressive therapy with prednisolone (PDN) 1 mg/kg/day (60 mg/day) was started, along with appropriate prophylaxis (proton pump inhibitor, calcium and vitamin D supplementation).

After eight weeks of PDN, only partial remission was achieved (proteinuria 2.1 g/24h, SAlb 3.1 g/dL). Cyclosporine was added (target trough 100–175 ng/mL), but the patient’s proteinuria, and hypoalbuminemia persisted and edema remained difficult to control. Prolonged steroid exposure led to Cushingoid features.

Given the persistent suboptimal response, RTX 1 g was administered two weeks apart, followed by gradual tapering of PDN. Prophylaxis with sulfamethoxazole-trimethoprim was started. Two months later, partial remission persisted (proteinuria 2.8 g/24 h, SAlb 3.3 g/dL) despite complete peripheral B-cell depletion (CD20 of 0 cel/µL), and blood pressure control within the aim. A sodium–glucose cotransporter-2 (SGLT2) inhibitor (dapagliflozin 10 mg daily) was added at this stage but was discontinued approximately 14 months later because of recurrent urinary tract infections (UTI). Six months after the initial RTX infusion, partial remission persisted (proteinuria 1.1 g/24 h, serum albumin 3.1 g/dL). Peripheral B-cell repopulation (CD20 1.1 cells/µL) led to a repeat RTX infusion (0.5 g). One month later, no further improvement was observed despite complete peripheral B-cell depletion.

Given persistent proteinuria despite adequate immunosuppression and peripheral B-cell depletion, a second renal biopsy was performed approximately one year after presentation, including evaluation by electron microscopy. This biopsy showed 25 glomeruli, again demonstrating a single FSGS NOS lesion, preserved architecture in the remaining glomeruli, mild IFTA (around 10%) and mild arteriosclerosis, and no significant immune deposits on immunofluorescence. Electron microscopy demonstrated diffuse podocyte foot process effacement (>90%), glomerular basement membrane with areas of reduced thickness (21–118 nm), showing focal irregularities and regions of increased thickness, slight mesangial and glomerular basement membrane sclerosis, and no immune-type deposits or organized structures were observed.

These results excluded significant chronicity, suggesting ongoing immunologically active disease rather than adaptive FSGS. Comprehensive genetic testing was performed using next-generation sequencing–based whole-exome sequencing, including genes associated with hereditary glomerular diseases and podocytopathies (notably COL4A3, COL4A4, COL4A5, and key podocyte-related genes such as *NPHS1, NPHS2, WT1, INF2, ACTN4*, and *TRPC6*) (see **Supplementary Material S1**). No pathogenic or likely pathogenic variants explaining the phenotype were identified, and variants of uncertain significance without phenotypic correlation were not reported. In conjunction with the absence of family history and lack of hematuria, a genetic etiology—including thin basement membrane disease—was considered unlikely, further supporting the diagnosis of primary FSGS.

**Figure 1:**
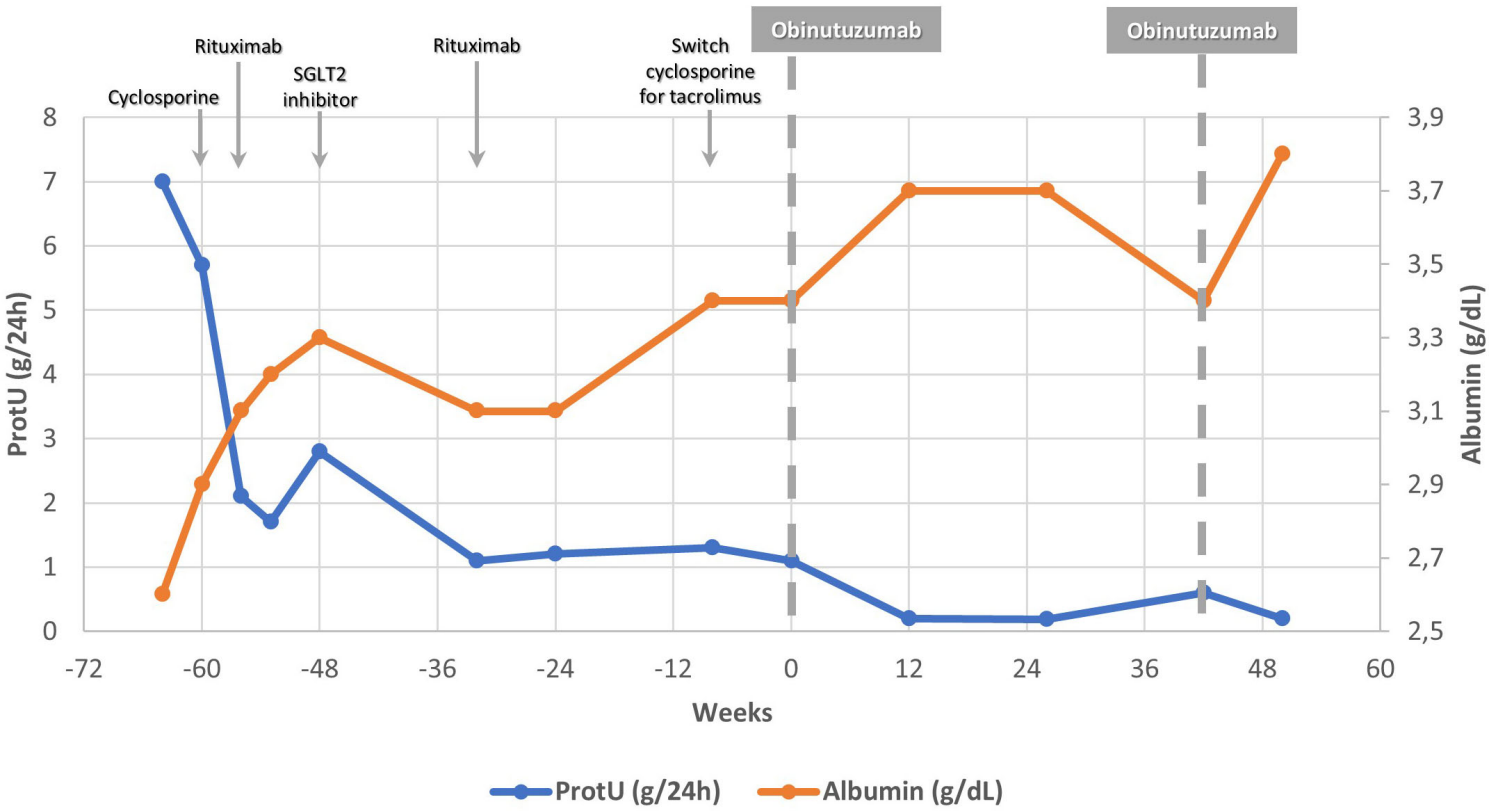
Changes of proteinuria and serum albumin levels before and after obinutuzumab administration. Shown are the 24-hour urinary protein excretion (ProtU, blue line; left y-axis) and serum albumin concentration (orange line; right y-axis) over time.

By this time, a switch to tacrolimus was attempted (target trough levels 5–8 ng/mL) due to cosmetic adverse effects. Due to persistent proteinuria with hypoalbuminemia despite initial peripheral B-cell depletion, RTX incomplete response was considered, and OBZ therapy was subsequently requested and approved. Thus, six months after the last RTX infusion, while on low-dose prednisolone (5 mg/day) and tacrolimus, with proteinuria of 1.1 g/24 h, SAlb 3.4 g/dL, and a CD20 count of 12.5 cel/µL the patient received OBZ 1 g administered twice, two weeks apart. Premedication included paracetamol, methyprednisolone and antihistamine, and no infusion-related reactions occurred. Prophylaxis with sulfamethoxazole-trimethoprim was continued. Two months after OBZ, the patient achieved complete remission, with a proteinuria of 0.2 g/24 h, and a SAlb of 3.7 g/dL, accompanied by sustained peripheral B-cell depletion (CD20 of 0 cel/µL), **Figure 1**. Corticosteroids and tacrolimus were gradually tapered until discontinuation. Complete remission persisted at 9 months of follow-up with preserved renal function, **Table 1**. No infectious complications were observed following OBZ, apart from urinary tract infections temporally related to prior SGLT2 inhibitor use, which resolved after drug withdrawal. The patient had documented immunity to hepatitis B from childhood vaccination. Hematologic parameters remained stable, and serum immunoglobulins (IgG and IgM), monitored every six months, remained within normal limits. No clinically relevant safety signals were identified.

**Table 1:** Temporal evolution of the clinical course, therapeutic interventions, and corresponding renal parameters relative to obinutuzumab administration (Week 0).

Weeks	Serum creatinine	eGFR	ProtU (g/24h)	UPCR (g/g)	SAlb (g/dL)	Prednisolone	Ciclosporin^1^	Tacrolimus^2^	Rituximab	Obinutuzumab	Dapagliflozin
-64	0.80	99,71	7.0	4.1	2,6	60 mg/day					
-60	0.77	103,74	5.7	Ø	2,9	60 mg/day	**Start**				
-56	0.99	76,73	2.2	Ø	3,1	40 mg/day	On target		1 g twice within 2 weeks		
-52	1.08	69,13	1.7	1.4	3,2	20mg/dia	On target				
-48	0.96	79,62	2.8	1.8	3,3	10 mg/day	On target				**Start 10mg/day**
-40	1.01	74,91	2.5	1.5	3.2	5 mg/dia	On target				10mg/day
-32	0.91	84,90	1.1	0.8	3,1	5mg/day	On target		0,5 g		10mg/day
-24	0.92	83,79	1.2	0.7	3,1	5mg/day	On target				10mg/day
-20	0.85	92,14	1.4	0.8	3.1	5mg/day	On target				10mg/day
-16	0.95	80,63	Ø	Ø	3.2	5mg/day	Ø				10mg/day
-12	Ø	Ø	Ø	Ø	Ø	5mg/day	Ø				10mg/day
-8	0.93	82,20	1.3	Ø	3,4	5mg/day	**On target>Stop**	**Start**			10mg/day
-4	0.78	101,52	1.1	Ø	3.3	5mg/day		Inferior to target			10mg/day
0	0.73	109,92	1.1	0.9	3,4	5 mg/day		Inferior to target		1 g twice within 2 weeks	10mg/day
4	Ø	Ø	Ø	Ø	Ø	5 mg/day		Inferior to target			10mg/day
8	0.79	99,98	0.8	Ø	3.7	2,5mg/day		Inferior to target			**Stop**
12	0.79	99,98	0.2	Ø	3,7	2,5mg/day		Inferior to target			
16	0.73	109,92	Ø	0.4	3.6	2,5mg/48h		On target			
20	Ø	Ø	Ø	Ø	Ø	**Stop**		Ø			
24	0.78	101,52	0.2	0.1	3,6			**On target>Stop**			
32	Ø	Ø	Ø	Ø	Ø						
42	0.76	104,08	0.6	0.1	3,4					1 g	
50	0.75	105,75	0.2	0.04	3,8						

¹Target trough blood levels 100–175 ng/mL; ²Target trough levels 5–8 ng/mL; tacrolimus doses were adjusted when trough levels were below target; Ø Value not measured at the specified time point; eGFR, estimated glomerular filtration rate (CKD-EPI); ProtU, 24-hour proteinuria; SAlb, serum albumin; UPCR, urine protein-to-creatinine ratio. Bold values indicate therapeutic interventions (initiation or discontinuation).

A repeat administration of OBZ (1 g) was performed 10 months later due to B-cell repopulation, rising proteinuria (0.6 g/24 h), and mild hypoalbuminemia (serum albumin 3.4 g/dL), successfully re-inducing complete remission (proteinuria 0.2 g/24 h, serum albumin 3.8 g/dL. Previous studies have demonstrated an association between B-cell recovery and impending relapse in podocytopathies (11). Accordingly, retreatment was undertaken as a pre-emptive strategy to prevent a full nephrotic relapse, particularly given the patient’s young age and the established association between repeated relapses and progressive glomerulosclerosis.

## 3 Discussion

This case highlights the therapeutic challenge posed by podocytopathies such as MCD and FSGS, particularly in patients who are frequently-relapsing, steroid dependent or resistant to standard immunosuppressive regimens. These patients are frequently exposed to significant treatment-related toxicity, increased morbidity, and impaired quality of life.

We report the case of a young woman with primary FSGS who achieved only partial remission after standard high-dose corticosteroid therapy. This led to significant steroid-related adverse effects, including Cushingoid features, markedly impairing her quality of life. Subsequent treatment with a calcineurin inhibitor resulted in minimal clinical benefit and was associated with cosmetically distressing adverse effects, in addition to the well-recognized risk of long-term nephrotoxicity. Subsequently, RTX was attempted but also failed to achieve complete remission.

RTX resistance/inadequate response is generally defined as a failure to achieve complete (urinary protein excretion <0.3 g/day) or partial (proteinuria <3.5 g/day with ≥50% reduction from baseline) remission of the nephrotic syndrome, with persistent hypoalbuminemia (SAlb <3.5 g/dL) (12). In the present case, although RTX was followed by some reduction in proteinuria and improvement in serum albumin, proteinuria never decreased below 1 g/24 h and serum albumin failed to normalize, as shown in **Figure 1**. Accordingly, this clinical course is best characterized as an unsatisfactory or incomplete response to RTX, consistent with definitions used in prior studies. Achieving complete remission in primary FSGS is associated with significantly improved long-term kidney survival compared with partial remission or persistent nephrotic-range proteinuria. Previous studies have demonstrated that patients achieving complete remission exhibit markedly lower rates of progression to end-stage kidney disease, whereas partial remission confers only intermediate protection (13, 14).

Several mechanisms have been proposed to explain inadequate response to RTX. This inadequate response may arise from underdosing or impaired bioavailability due to internalization and degradation of type I anti-CD20 antibodies (6, 15). In addition, in nephrotic syndrome, RTX can bind to albumin and be lost in the urine, leading to reduced serum concentrations. Patients with severe nephrotic syndrome (baseline serum albumin <2.25 g/dL) are particularly prone to exhibit undetectable RTX levels 3 months after infusion (15). Although serum RTX concentrations could not be measured at our institution, RTX was administered after partial remission had been achieved (proteinuria 1.1 g/24 h), a context in which substantial urinary loss is less likely. In addition, peripheral B-cell subsets were monitored and demonstrated sustained circulating B-cell depletion following RTX administration, indicating preserved pharmacodynamic activity despite an incomplete clinical response. Collectively, these observations make insufficient drug exposure a less likely explanation for the lack of complete remission.

Another proposed mechanism of inadequate response to RTX is the development of anti-RTX antibodies, reported in up to 23–43% of treated patients, which may neutralize drug activity, accelerate B-cell reconstitution, and increase relapse risk (15, 16). Although this testing was unavailable at our institution, several indirect observations make this mechanism less likely in the present case. The patient had no prior exposure to anti-CD20 therapy, anti-RTX antibodies are typically detected several months after initial exposure, and sustained peripheral CD20-positive B-cell depletion was documented following RTX administration. Taken together, these findings argue against a clinically relevant neutralizing antibody response in this patient (17).

An alternative explanation for persistent proteinuria is irreversible glomerular injury. Distinguishing between immunologic resistance and chronic structural damage is critical, as treatment strategies differ. In such cases, a repeat kidney biopsy is valuable to assess ongoing disease activity versus chronicity (15). In our patient, the second biopsy revealed minimal chronic changes, suggesting active disease rather than irreversible scarring. Genetic testing was also performed to exclude hereditary podocytopathy, with negative results.

Another potential mechanism of inadequate response to RTX involves incomplete B-cell depletion in secondary lymphoid organs. Translational studies in hematologic diseases, lupus nephritis and other immune-mediated kidney diseases have demonstrated persistent tissue-resident and memory B cells after RTX therapy, providing biologic plausibility for ongoing disease activity despite apparent pharmacodynamic success in blood (18). Incomplete B-cell depletion may result in persistent autoreactive memory B cell clones that may continue to proliferate and differentiate into antibody-secreting cells, leading to ongoing disease activity (9). Persistence of autoreactive memory B-cell clones within lymph nodes and spleen may therefore sustain pathogenic immune activity, even in the absence of circulating CD20-positive B cells. In our patient, although complete peripheral B-cell depletion was documented, the authors believe that persistent disease activity suggests that depletion of tissue-resident B cells may have been insufficient. OBZ has been shown to induce deeper and more sustained B-cell depletion than RTX, particularly within secondary lymphoid tissues, likely reflecting its distinct type II anti-CD20 properties and enhanced antibody-dependent cellular cytotoxicity (6, 7). Moreover, its humanized structure confers lower immunogenicity and reduced likelihood of anti-drug antibody formation.

In the present case, OBZ induced complete remission within two months, with normalization of serum albumin, sustained B-cell depletion, and no infusion-related or infectious complications. This clinical outcome suggests that OBZ may have overcome RTX inadequate response through more potent and comprehensive B-cell depletion, reinforcing that therapeutic success is not solely dependent on circulating B-cell depletion, but rather on effective elimination of tissue-resident pathogenic B-cell populations. No lymph node biopsy was performed, as the patient demonstrated clear clinical improvement and the potential risks of an invasive procedure were considered to outweigh the anticipated diagnostic benefit.

Emerging data from kidney-disease settings support the biologic plausibility of OBZ in refractory immune-mediated nephropathies. Studies in end-stage renal disease and transplant candidates have demonstrated rapid, profound, and durable depletion of B-cell subsets extending beyond peripheral blood into tissue compartments, with tissue-resident B cell populations assessed directly in lymphoid tissues, supporting the concept that this type II anti-CD20 antibody achieves deeper compartmental depletion than can be inferred from blood counts alone (7). In parallel, lupus nephritis trials have demonstrated improved renal outcomes and robust B-cell depletion with OBZ added to standard therapy, reinforcing the biologic rationale for a more potent and sustained B-cell–depleting strategy when RTX is insufficient (18).

Evidence for OBZ in podocytopathies remains limited to small pediatric (19) and adult series (20–24) and isolated case reports (25–27), with substantial clinical heterogeneity. Reported cases range from frequently relapsing or immunosuppression-dependent disease to RTX-resistance, with variable prior exposure to corticosteroids, calcineurin inhibitors, and anti-CD20 therapy. Despite that, across these reports, remission rates appear encouraging, **Table 2**. However, follow-up durations are generally short, sample sizes are small, the durability of response remains uncertain, and retreatment strategies were not standardized. Consequently, the role of obinutuzumab as a long-term maintenance therapy in FSGS remains to be clearly defined.

**Table 2:** Summary of published case series evaluating obinutuzumab in resistant or dependent podocytopathies.

Variables	Dossier et al., 2023 (19)	Zand et al., 2024 (22)	Jin et al., 2024 (20)	Lin et al., 2025 (21)	Angeletti et al., 2025 (23)	Chen et al., 2025 (24)
Sample Size	41	20	6	11	6	5
Age (years)	4 (IQR, 3–6)	$\bar{\text{x}}$: 45.3 ± 17.5	30.4 (IQR 18.8–72.1)	26.0 (IQR 21.0–35.0)	14.5 (IQR 12.0–17.0)	$\bar{\text{x}}$: 26.2 ± 14.9
Setting	IMS-resistant/ dependent	IMS-resistant/ dependent	IMS-resistant/ dependent	IMS-resistant/ dependent	IMS-resistant	RTX-resistant
Follow-up (months)	43 (IQR 36–52)	12	$\bar{\text{x}}$: 12.53 ± 1.97	17 (IQR 12–22)	12	12
Previous Treatments		Not specified				
- GC	100%		100%	81.8%	100%	100%
- RTX	100%		16.7%	45.5%	100%	100%
- Ofatumumab	17.0%		-	-	-	-
- CNI	43.0%		33.3%	54.5	83.3%	60.0%
- CYP	10.0%		16.7%	-	-	-
- Mycophenolate	78.0%		-	-	33.3%	-
- Others	63.0%		33.3%	-	-	-
Obinutuzumab	Single infusion 300mg/1.73m^2^	1g x 2 doses, 2 weeks apart^1^	1g x 2 doses, 2 weeks apart ^2^	1 g^3^	1g x 2 doses, 2 weeks apart	1 g^4^
Patients achieving CR/PR	92.0%	40.0%	100%	100%	100%	100%
Relapse-free during follow-up	68.0%	40.0%	100%	81.8%	100%	80.0%
Adverse Events						
- Infusion-related reaction	12.0%	35.0%	-	18.2%	-	-
- Infections not requiring hospitalization	-	35.0%	-	9.1%	16.7%	-
- Infections requiring hospitalization	2.4%	-	-	9.1%	-	-
- Others	29.3%	15.0%	-	9.1%	-	-

^1^ Other administrations every 6 months; ^2^ One patient received an additional dose at 6 months; ^3^ The initial obinutuzumab course was continued until peripheral CD19^+^ B-cell counts reached 0 cells/µL, thus additional 1 g doses were administered every 2 weeks until complete B-cell depletion was achieved.; ^4^ Additional administrations according to physician discretion; CNI, calcineurin inhibitor; CR/PR, complete remission/partial remission; CYP, cyclophosphamide; GC, glucocorticoids; IMS, immunosuppression; IQR, interquartile range; RTX, rituximab.

Although peripheral CD20-positive B-cell counts do not directly reflect tissue depletion, their reconstitution may serve as a pragmatic surrogate marker of broader immune reconstitution and help guide retreatment timing, supported by previous studies demonstrating an association between CD19/CD20+ B-cell recovery and impending relapse in podocytopathies, with B-cell reappearance often preceding clinical relapse by several weeks (11).

Our case adds to the emerging evidence that OBZ may represent a safe and effective alternative in this subset of patients, potentially minimizing the cumulative toxicity of prolonged corticosteroid and calcineurin inhibitor exposure. Moreover, it underscores the clinical importance of pursuing complete remission, rather than accepting partial remission in the presence of persistent disease activity, to optimize long-term outcomes and quality of life. In this context, the ability of OBZ to induce complete rather than partial remission may carry meaningful prognostic implications.

These observations suggest that larger-scale studies are required to evaluate the effect of OBZ in refractory podocytopathies such as MCD and FSGS, to better establish its role in these conditions.

## Data Availability

The original contributions presented in the study are included in the article/**Supplementary Material**. Further inquiries can be directed to the corresponding author.
