# Supplementary material for: Successful use of obinutuzumab in focal segmental glomerulosclerosis with inadequate response to rituximab: a case report
Source: Front Nephrol. 2026 Mar 3;6:1772736. doi: 10.3389/fneph.2026.1772736 (PMC12992063; doi:10.3389/fneph.2026.1772736)
Supplement: Supplementary file 1 [file Table1.docx]

**Supplementary Material:**

**S1. Full list of analyzed genes:**

**Glomerular basement membrane / COL4-related nephropathies:** COL4A1, COL4A3, COL4A4, COL4A5

**Podocyte-associated genes (FSGS / podocytopathies):** ACTN4, ANLN, ARHGAP24, ARHGAP26, CD2AP, DLC1, FAT1, FAT4, INF2, KANK1, KANK2, KANK4, LAMA5, LAMB2, LMX1B, MAGI2, MYH9, MYO1E, NPHS1, NPHS2, PLCE1, PTPRO, TRPC6, WT1

**Complement system / thrombotic microangiopathy:** C1QA, C1QB, C1QC, CFB, CFH, CFHR1, CFHR3, CFHR5, CFI, DGKE, THBD

**APOL1 and risk alleles:** APOL1

**Ciliopathies / nephronophthisis-related genes:** AHI1, ANKS6, ARL3, BBIP1, BBS1, BBS4, BBS5, BBS7, BBS9, BBS10, BBS12, C2CD3, CEP41, CEP55, CEP83, CEP104, CEP120, CEP164, CEP290, CPLANE1, CSPP1, DZIP1L, HYLS1, IFT27, IFT43, IFT74, IFT122, IFT140, IFT172, INVS, IQCB1, KIAA0586, KIAA0753, LZTFL1, MKS1, NEK8, NPHP1, NPHP3, NPHP4, OFD1, SDCCAG8, SCLT1, TCTN2, TCTN3, TMEM67, TMEM107, TMEM138, TMEM216, TMEM231, TMEM237, TTC8, TTC21B, WDPCP, WDR19, WDR35, WDR73, ZNF423

**Tubulointerstitial kidney diseases / electrolyte disorders:** ATP6V0A4, ATP6V1B1, BSND, CLCN5, CLCNKA, CLCNKB, CLDN16, CLDN19, CNNM2, FXYD2, GATM, KCNJ1, KCNJ10, NR3C2, SCNN1A, SCNN1B, SCNN1G, SLC12A1, SLC12A3, SLC22A12, SLC26A1, SLC34A1, SLC34A3, SLC4A4, SLC7A7, SLC7A9, SLC9A3R1, UMOD, WNK1, WNK4

**Congenital anomalies of the kidney and urinary tract (CAKUT):** BMPER, CHD7, EYA1, FRAS1, FREM1, FREM2, GATA3, GREB1L, HNF1B, JAG1, NOTCH2, PAX2, ROBO2, SIX1, SIX5, TBX18, TFAP2A, WNT4, WNT5A

**Cystic kidney diseases:** GANAB, PKD1, PKD2, PKHD1, PRKCSH, SEC61A1, SEC63

**Mitochondrial / metabolic nephropathies:** BCS1L, COQ2, COQ6, COQ8B, CPT2, FAH, G6PC, HOGA1, LDHA, LCAT, MMACHC, MUT, PDSS1, PDSS2, RMND1, UQCC2

**Syndromic / multisystem disorders with renal involvement:** ALMS1, ARMC9, CISD2, DNAJB11, EIF2AK3, ELP1, FAN1, FANCA, HPS1, HPS4, LRP2, MNX1, OCRL, PHF6, PIGA, VPS33B, VIPAS39

**Other genes associated with hereditary kidney disease:** ABCC6, ACE, ACTB, ADA2, ADAMTS13, ADAMTS9, ADCY10, AGT, AGTR1, AGXT, ALG1, ALG8, ALG9, ALPL, ANOS1, AP2S1, APOA1, APOE, APRT, AQP2, ATP7B, AVPR2, B2M, BNC2, CD46, CDC73, CDKN1C, CRB2, CTNS, CUL3, CYP11B1, CYP24A1, DCDC2, DCHS1, DHCR7, DNASE1L3, DSTYK, EBP, EMP2, ENPP1, FAM20A, FGF20, FGF23, FIBP, FN1, GALNT3, GCM2, GLA, GLI3, GLIS3, GNA11, GPC3, GRHPR, GRIP1, HGD, HPRT1, HPSE2, HSD11B2, ITGA3, ITGA6, ITGA8, KAT6B, KCTD1, KLHL3, LAGE3, LRP4, MAGED2, MAPKBP1, MEFV, MOCOS, NSDHL, NUP85, NUP93, NUP107, NUP133, NUP160, NUP205, OSBPL9, PDE6D, PEX6, PHGDH, PLG, PMM2, PRPS1, REN, RPL26, RPS26, SALL1, SALL4, SARS2, SCARB2, SGPL1, SI, SLC1A1, SLC2A2, SLC2A5, SLC3A1, SLC4A1, SLX4, SMARCAL1, SOX18, TBC1D8B, TMEM107, TPSAB1, TRAF3IP1, TRIM32, TSC1, TSC2, TXNDC15, VHL, XDH, XPNPEP3, XPO5, YRDC.
